# Editorial: Insights in biosafety and biosecurity 2024/2025: novel developments, current challenges, and future perspectives

**DOI:** 10.3389/fbioe.2026.1855658

**Published:** 2026-05-19

**Authors:** Segaran P. Pillai, Stephen A. Morse, Lela Bakanidze

**Affiliations:** 1 Department of Health and Human Services, Food and Drug Administration, Office of the Commissioner, Silver Spring, MD, United States; 2 IHRC, Inc., Atlanta, GA, United States; 3 National Center for Disease Control and Public Health (Georgia), Tiblisi, Georgia

**Keywords:** biosafety and biosecurity, dual use research of concern, laboratory quality management system, risk assessments, synthetic biology and AI

The increased accessibility of biotechnological tools and the growing complexity of laboratory research have introduced new challenges to the safe and secure management of biological materials. The concepts of biosafety and biosecurity have therefore become central to the governance of life science research. Biosafety primarily involves laboratory practices that aims to protect laboratory personnel, communities, and the environment from accidental harm, while biosecurity focuses on mitigating unauthorized access, loss, theft, misuse, deliberate threats, bioterrorism, agroterrorism, and the dual-use potential of scientific research.

In 2022, Frontiers developed the Research Topic “*Insights in Biosafety and Biosecurity: Novel Developments, Current Challenges, and Future Perspectives.”* The objective was to explore how scientific research contributes to enhancing biosafety and biosecurity practices globally (Pillai and Raybould, 2023; Pillai and Morse, 2024).

For the current iteration of this Research Topic, there were 18 submissions of which 13 were accepted and published. The authors of the published submissions were from eight countries keeping with the global importance of this topic.

Morse identified the major biosafety and biosecurity challenges that have emerged from rapid advances in biotechnology. Global expansion of high-containment (BSL-3 and BSL-4) laboratories has increased risks of both accidental and intentional misuse. Uneven safety standards across countries have prompted calls for stronger, more consistent global biorisk management frameworks. At the same time, dual use research of concern (DURC) – including gain-of-function studies, synthetic virus reconstruction, and the rapid dissemination of preprints–poses growing challenges, particularly as more research occurs outside traditional government-funded systems. Advances in synthetic biology further expand the ability to re-create pathogens, enhance microbes, and produce harmful biological materials, while artificial intelligence (AI) amplifies these capabilities. These trends underscore the need for updated biosecurity measures, including expanded screening of sequences of concern and stricter verification of end users.

AI-driven protein design offers transformative potential for bioengineering and biotechnology (Wheeler) but also introduces significant biosecurity risks. These include the possibility of AI-enabled development of harmful biological agents and the misuse of increasingly accessible design tools. Addressing these challenges will require coordinated policy frameworks, technical safeguards, and community-based governance approaches that reduce risks while enabling responsible innovation.

Greene et al. investigated variability in reviewing life science projects identified as potential DURC where U.S. policy recommends risk assessments to guide management strategies. Little is known about how consistent assessments were applied or how reviewers differ. In their study, 18 expert reviewers and 49 synthetic biology students evaluated four real-word projects using a modified U.S. government DURC framework. Results showed wide variation in perceived risk and recommended strategies, with expert ratings differing by up to four out of five levels. This inconsistency suggests the need for standardized methods to improve agreement, accuracy, and cost-effectiveness.

Gillum et al. examined biosafety and biosecurity practices among professionals conducting research on enhanced Potential Pandemic Pathogens (ePPPs) and DURC across U.S. sectors. A survey of 541 respondents found that institutions with larger teams reported more research activity and stronger noncompliance reporting systems. Government organizations conducted more DURC, while public institutions reviewed more experiments outside the scope of U.S. DURC Policy than private for-profit groups. Variation in funding and policy implementation highlighted the need for adequate staffing, resources, tailored regulatory approaches, and stronger reporting systems.

The growth of biotechnology supports creating a National Biosafety and Biosecurity Agency (NBBA) in the U.S. to unify oversight (Gillum et al.). It would streamline regulations, and oversee high-risk research including DURC, ePPPs, nucleic acid synthesis screening, pathogen management and laboratory animal regulations.

Governance approaches for biotechnological risks generally follow three main approaches: the laissez-faire (“technology first”), preventive (“safety first” with quantitative analysis), and precautionary (“safety first” with multidimensional consideration) (Wang). Rapid advances in biotechnologies, and the uncertainty have made risk management more complex. The precautionary approach stands out for its focus on foresight, proportionality, and shared responsibility promoting awareness of risks, shifting the burden of proof, and aligning actions with potential risk. This framework supports responsible biotechnology while maintaining security and public trust.

Coronado et al. discussed the dual use dilemma in Latin America, where policies and institutional awareness remain limited. Addressing this requires robust biosafety and biosecurity systems, along with clear national and international regulations tailored to DURC. These frameworks should balance risk prevention with responsible innovation and open scientific exchange.

The emergence and reemergence of infectious diseases across human, animal, and environmental sectors have expanded BSL-3 laboratories in India under the National One Health Mission (Patil et al.). Currently, no standardized tool exists to evaluate biosafety, biosecurity, competency, or sample management. To address this, a comprehensive assessment tool was developed to evaluate BSL-3 laboratory performance. Aligned with International Health Regulations and the Global Health Security Agenda, it assesses laboratory preparedness for safe and prompt outbreak testing.

As technologies such as AI, machine learning, and advanced manufacturing evolve, laboratories must implement strong management and process improvement to maintain accuracy and reproducibility (Pillai and Fox). A Laboratory Quality Management System (LQMS) provides the structure to manage documentation, address performance gaps, and mitigate risks compromising data integrity and credibility. LQMS strengthens biosafety and biosecurity by ensuring all activities follow standardized, well-documented procedures promoting accurate results, safe handling of biological agents and accident prevention. Quality management also includes routine maintenance and calibration, reducing the risk of failures causing exposures or containment breaches.

Rapid advances in synthetic biology and technologies like AI, 3D printing, and nanotechnology pose biosecurity risks. Using a Delphi process (Sabra et al.), identified key biological threats and developed consensus-based policy recommendations. They proposed a hybrid governance framework, with four pillars: building awareness, enhanced biosecurity training, agile governance, and stronger international treaties such as the Biological Weapons Convention. Implementing these measures could curb misuse of emerging biotechnologies and strengthen global biosecurity.

Accidental laboratory-acquired infections (LAIs) in BSL-3/4 facilities increases the risk of releasing a pathogen, that could trigger a pandemic. Cohen et al. modeled 192 scenarios to assess outbreaks of ≥50 cases over 100 days. Frequent, sensitive testing reduced outbreak odds, while longer isolation delays weakened testing benefits. Rapid isolation of laboratory workers markedly lowered pathogen escape, with high-frequency testing offering the strongest protection.

Ottendorfer et al. shared that despite poliomyelitis elimination in the U.S, global resurgence and declining vaccination rates pose ongoing risks. U.S. laboratories are vital for vaccine production, diagnostics, and surveillance, making strict biosafety and containment essential. The U.S. National Authority for Containment (U.S. NAC) at the Centers for Disease Control and Prevention (CDC) oversees facilities, certifies biocontainment compliance, and has sharply reduced designated poliovirus facilities. Following the 2025 Executive Order withdrawing the U.S. from the World Health Organization (WHO), the U.S. NAC is developing a national certification framework based on international standards, independent of WHO systems.

Arthropod-borne viruses account for over 17% of global infections and cause more than 700,000 deaths annually as per the World Health Organization, 2020. Over 500 arboviruses have been identified, more than 150 causing human disease. To assess public health risks and guide investment, planning and preparedness Pillai et al. used multi-criteria decision analysis (MCDA) and a Decision Support Framework (DSF). A literature review of 54 arboviruses across 14 criteria for MCDA and nine for DSF informed the analysis. Both tools identified similar high-risk viruses, demonstrating that combining them strengthens risk assessment.

Despite numerous efforts, challenges have persisted in evaluating risk of synthetic biology and other biotechnologies, and digital biosecurity threats. Future research should explore integrated risk frameworks addressing both biological and informational risks. Research contributes to evidence-based policy and technical innovation and underpins a culture of responsibility that ensures scientific progress, benefits humanity, and minimizes biological risks.

The editors would like to thank the authors for their contributions to this Research Topic on the importance of biosafety and biosecurity to ensure the safe, responsible, and secure conduct of biological science research.
